# Enablers and barriers of male involvement in the use of modern family planning methods in Eastern Uganda: a qualitative study

**DOI:** 10.1186/s40834-023-00251-x

**Published:** 2023-10-16

**Authors:** Atkinson Tekakwo, Rose Chalo Nabirye, Ritah Nantale, Faith Oguttu, Brendah Nambozo, Solomon Wani, Milton W. Musaba, David Mukunya, Joshua Epuitai

**Affiliations:** 1 Department of Nursing, Faculty of Health Sciences, Busitema University, Mbale, Uganda; 2 Department of Community and Public Health, Faculty of Health Sciences Mbale, Busitema University, Mbale, Uganda; 3 Department of Obstetrics and Gynecology, Faculty of Health Sciences, Busitema University, Mbale, Uganda; 4 Busitema University Centre of Excellence for Maternal, Reproductive and Child Health, Mbale, Uganda; 5 Department of Research, Nikao Medical Center, Kampala, Uganda

**Keywords:** Male involvement, Family planning, Uganda

## Abstract

**Background:**

Male involvement plays a critical role in the utilization of various sexual and reproductive health services. We explored enablers and barriers of male involvement in the use of modern family planning methods in Eastern Uganda.

**Methods:**

This was a qualitative study in Mbale, Eastern Uganda done between November and December 2022. We conducted three group discussions comprising of four participants each, with male partners and eight key informant interviews with midwives. We followed a group discussion guide during the group discussions and an interview guide during the key informant interviews to explore enablers and barriers of male involvement in the use of modern family planning methods. All the interviews and group discussions were audio-recorded with permission from the participants, transcribed verbatim, and analyzed following thematic content analysis approach.

**Results:**

Two sub-themes emerged from the analysis; perceived enablers and barriers. The perceived enablers included positive attitude, subjective norms, need to support the woman, mutual consent, limited resources and expected benefits of reducing gender-based violence and sexually transmitted infections. Lack of male partner consent, busy work engagement, social stigma, religious prohibition, desire for many children and gender roles incompatibility hindered male partner involvement in family planning. Fear of side effects and misconceptions, unconducive hospital environment in form of mistreatment, family planning considered a female’s issue, and lack of consideration of male partner needs in family planning clinic were additional barriers to male involvement.

**Conclusion:**

Male involvement in family planning was related to positive attitude and subjective norms towards family planning, mutual consent, and recognition for limited resources to support a large family size. Lack of male partner approval, fear of side effects and misconceptions, unconducive hospital environment and social, cultural and religious prohibitions discouraged male partner involvement in family planning. Community based approaches to family planning sensitization, such as community education campaigns, may be an important step toward reducing barriers to male involvement in the use of modern family planning methods.

## Background

Male involvement in family planning refers to all organizational activities aimed at men as a discrete group with the objective of increasing the acceptability and use of family planning by either the woman or the male partner [1]. Family planning allows men, women, and couples to choose if and when to have children by way of intentionally delaying, spacing or limiting pregnancies [2]. Access to and use of modern family planning methods can improve health, socio-economic status and productivity [3]. Further, utilization of modern family planning methods reduces the risk of unintended pregnancies, sexually transmitted diseases and reduction in maternal and neonatal morbidity and mortality [4].

Despite the advances in the use of modern family planning methods globally, an estimated 214 million women in developing countries have an unmet need for modern family planning [5]. Furthermore, there is a variation in the use of modern family planning methods from 69% in Southeast Asia to 11% in Africa [6]. In Africa, the limited male involvement in the use of modern family planning methods is one of the contributing factors for the high unmet need for modern family planning methods [7]. Male involvement in family planning majorly includes encouraging the use of male family planning methods and expanding participation of men in decision-making [2]. In addition, male involvement improves uptake of family planning methods by women through spousal communication and decreasing opposition thus decreasing incidences of method discontinuation [8].

Uganda has the third fastest growing population in the world [9] with a total fertility rate of 5.4 which is the fifth highest in the world [10, 11]. The modern contraceptive use in Uganda is at 35% [11] and unmet need for family planning estimated at 33% [11]. The low contraceptive use and increased fertility rate contributes to the rapid population growth in the country [11, 12]. Rapid population growth increases the burden on the already limited services and resources in the country [13]. The government of Uganda has made many directed initiatives and improvement in the distribution, access and use of modern family planning methods in all the health facilities in the country [14]. Encouraging active male involvement in the use of family planning, would address the challenge of unmet need among women [15]. However, there is a dearth of data on enablers and barriers of male involvement in the use of modern family planning methods in Uganda. We explored enablers and barriers for male involvement in the use of modern family planning methods in Eastern Uganda.

## Methods

### Study design

We conducted a qualitative study to explore the deeper meanings and perceptions regarding male involvement in the use of modern family planning methods. Data collection was conducted between November and December 2022.

### Study setting

The study was conducted at Mbale Regional Referral Hospital, and Namatala Health Center IV in Mbale City, Eastern Uganda. According to the Uganda Demographic and Health Survey of 2016, modern family planning use among women in Bugisu region was at 43% [11]. Mbale Regional Referral Hospital serves the Elgon region which is a population of about 4 million people from 14 districts in Eastern Uganda. Namatala Health Center IV is a level four health facility within Mbale City. The two health facilities provide all modern family planning methods including oral contraceptive pills, implants, injectables, contraceptive patch and vaginal ring, intrauterine device (IUD), female and male condoms, female and male sterilization, vaginal barrier methods (including the diaphragm, cervical cap and spermicidal agents). According to the records on the use of family planning methods at the two hospitals, the most commonly used methods are injectable contraceptives and oral contraceptive pills.

### Study population and sampling technique

The study population included male partners and midwives who were involved in provision of modern family planning methods. Male partners were recruited from the community in Namatala informal settlements to participate in group discussions, while midwives were recruited as key informants from the family planning clinics of the aforementioned health facilities. We recruited eight midwives including three from Mbale regional referral hospital and five from Namatala Health Center IV.

Purposive sampling technique was used to select the study participants for the study. The criteria for purposive sampling included characteristics such as being in an intimate relationship, male partners whose partners were or were not using modern family planning methods, had leadership roles in the community and male partners who had more than one partner. Purposive sampling enabled selection of participants who would provide in-depth insight regarding male involvement in family planning. The sample size was determined basing on the principle of data saturation. In this study, saturation was reached after three group discussions and eight key informant interviews.

### Data collection tool and procedure

We used group discussions and key informant interviews for data collection. Each group discussion had four participants. We followed a group discussion guide during the group discussions and an interview guide during the key informant interviews. The group discussion guide used in the group discussions included questions such as beliefs and thoughts about family planning, importance of family planning, reasons for male involvement and against male involvement in family planning, ways in which male partners can get involved in family planning. The interview guide used in the key-informant interviews explored perceptions of midwives regarding the enablers and barriers to male involvement including reasons for and against male involvement in family planning. Data were collected by trained research assistants who were natives of the language. Group discussions were conducted in Lumasaba, the local dialect and key informant interviews were conducted in English language. The interviews and group discussions lasted about 40 to 50 minutes. All interviews and group discussions were audio recorded with permission from the participants.

### Data analysis and rigor

The transcripts were transcribed verbatim.The transcripts in the local language were translated to English by a native of the language. We used thematic content analysis to analyze the data [16]. The analysis followed a five-step process. First, we read through the transcripts and became familiar with the data. Secondly, we organized data in a meaningful way and generated the initial codes. Once the data had been sufficiently coded and saturation reached, we identified themes. We then reviewed and modified themes and summarized our findings. Data analysis was conducted by AT, JE & RCN.

Rigor and trustworthiness of the data was maintained through triangulation of group discussions and key informant interviews. The themes and codes were not pre-selected which ensured that the interpretation of the transcripts was grounded in the data. The use of trained research assistants, nursing students who were familiar with family planning, and natives of the language ensured the credibility of the data.

## Results

### Description of the participants

We conducted three group discussions among men and eight key informant interviews with midwives. Nine of the 12 participants in the group discussions were married, while the remaining three were co-habiting. Four of the participants in the group discussions were Catholics, five were Muslims and one were Anglican and two were Pentecostals. Ten of the participants were in self-employment with secondary level of education, while the remaining two were in formal employment with university level of education. The average age of male participants in the group discussion were 29.6 (standard deviation = 4.8), while the average age of the key informants was 33 (standard deviation = 5.6). The key informants were midwives of which three were of Certificate level of education and five were of Diploma level of training.

### Perceived enablers of male involvement

Perceived enablers for male involvement in the use of modern family planning methods included positive attitudes, subjective norms, motivation to show support for the woman, mutual consent to participate in family planning and limited financial resources (Table 1).

**Table 1:** Perceived enablers and barriers for male involvement in the use of modern family planning methods

Quotes	Categories	Sub themes
*“.It is a good thing. I would actually encourage everybody to participate”*	Positive attitude	Perceived enablers of male involvement in the use of modern family planning methods
*“when I see you going to the health centre with your wife basically to access FP services, definitely I will also be motivated”*	Subjective norms	
*“Now for the ones who want to participate, they actually do it because they want to show their partner that they care and [that] they are supportive”*	A sign of support	
*“Yes, men can actually participate for as long as it is agreed upon by both the man and the woman”*	Mutual consent	
*“I think it is a good thing to get involved in the partner’s use because…., you don’t have enough resources to look after them"*	Limited resources	
*“.…. because it helps us to reduce the gender-based violence”* *“Like if we encourage men also there will be a reduction in the STI…”*	Benefits of male involvement	
		Perceived barriers of male involvement in the use of modern family planning methods
*“But now if the man and the woman are not agreeing,….they go and get the services behind their man’s back…. So, such a man may not support the wife…”*	Lack of male partner consent	
*“They always take that FP are women’s thing. They always think that it is the women to do it.”*	Family planning considered a woman’s issue	
*“Those ones who don’t escort maybe because….they are always engaged”*	Busy work schedules	
*“Now also that stigma….”* *“….So, he doesn’t want to be perceived as a weak man, so definitely he cannot get involved”* *“The society views…like the woman is controlling him…”*	Social pressure	
*“Then another factor may also be the religious prohibition……”*	Religious prohibition	
*“But to me, I will say, a man escorting the wife to the clinic? It means the man has totally failed to manage his own family or home….”*	Gender roles incompatibility	
*“Some men may not want to participate…. the desire to have children”*	Desire for children	
*“the reason why the men don’t want to participate is that, women experience side effects, more so over bleeding”*	Fear of side effects	
*“I am negative about Uganda’s family to be specific because the agenda has gone wrong”*	Misconceptions	
*“I think it is because of inadequate information about FP*	Inadequate information	
*“….some of them have no access to the service….FP methods or services are not very near in these village hospitals.”* *“are not really settled. You just keep walking up and down.”*	Inaccessible family planning services	
*“Then the ones who don’t want to participate… because maybe….they were talked to rudely by the nurses at the FP….”*	Mistreatment in the health facility	
*“…some men just fear to go to the hospital.”* *“As a man you feel like out of place because all this line is just full of females. The men are really uncomfortable.”*	Unconducive hospital setting	

### Positive attitudes (n = 5)

Positive attitudes towards family planning encouraged male partner involvement in the use of family planning. Male partners acknowledged that family planning removes worries of unwanted pregnancy, allows spacing of children, and were not associated with serious side effects as it was widely purported.

*“Generally, I think FP is not a bad thing especially if we are sensitized about it and we are made to know about those things in details and how we can benefit from them. It is a good thing. I would actually encourage everybody to participate” (Married man, Group discussion 1)*.

*“When my partner is using FP, I feel relaxed because now I am not worried about pregnancies even though the other child is still small. I am not worried about the pregnancy” (Married man, Group discussion 3)*.

*“Now like condoms, I really don’t see much dangers in the use of condoms though a man may have a perception that he doesn’t enjoy sex when using condoms but I don’t see that as a danger especially when you want to work on child spacing” (Married man, Group discussion 1)*.

### Subjective norms (n = 1)

Male involvement was seen to be positively influenced by the behaviors of their peers. Male partners who escorted their partners to family planning unit were perceived to encourage other male partners to participate in family planning.

*“So, men who actually go or who escorts their wives to these health facilities purposively to access these FP methods, they are actually encouraging other men. They show good examples to these other men….They are actually increasing acceptability and the use of FP….when I see you going to the health centre with your wife basically to access FP services, definitely I will also be motivated” (Married man, Group discussion 2, mechanic)*.

### A sign of support (n = 4)

Male partners were motivated to participate in family planning because of the need to show support and care to the woman.

*“Now for the ones who want to participate, they actually do it because they want to show their partner that they care and [that] they are supportive” (Married man, Group discussion 3)*.

*“…… your wife will know that you are in full support of it… the only way of showing that you are in support of it is by going with her to the family planning clinic… and that I think is one way of showing her love and even participating….”(Mid-wife, KII 2)*.

Besides a sign of support, some of the men were involved in family planning out of their desire to learn more about family planning

*“I can say that as a man, to know more about FP, you have to go with her. This will help you get more information about FP from a doctor or someone who is there who know more about FP so that he can tell you more about FP so that you can”* (*Married man, Group discussion 2*).

### Mutual consent (n = 6)

Most of the male partners were concerned about the side effects that might occur from the use of family planning. As a result, male partners believed that collective decision making would enable them to carefully evaluate the side effects before choosing any family planning method. The decision to use family planning was thought to transcend the individual which necessitated involvement of the whole family. Male partners were willing to participate and get involved in family planning use as long as they mutually agreed as a couple to use family planning. Mutual consent was thought to avoid marital conflicts in case of any side effects

*“Yes, men can actually participate for as long as it is agreed upon by both the man and the woman. Of course, the basic here is we need to first agree on what to do like on the FP to use and we support each other”(Married man, Group discussion 2)*.

*“That [mutual agreement] is what most men need because in most cases the women choose by themselves without the knowledge of their husbands. So, if we can sit down as a family and we decide then it is okey….Because I feel like these FP things are not only for two people but is for the whole community”( Married man, Group discussion 1)*.

### Limited resources to support the family (n = 12)

Male participation in the use of family planning methods was related to need to limit the size of the family, and ultimately, reduce family expenditures. Large families were thought to be unsustainable with the rising costs of living, financial constraints, and limited resources including space to accommodate a big family. However, motivation for a small family size was seen as fearing responsibilities which was perceived to be untenable in the long-term.

*“I think it is a good thing to get involved in the family planning use because you cannot continue to have children every year and yet you are not able to take care of them especially one, you don’t have enough resources to look after them because more children mean more mouths to feed, more bodies to clothe, more people to shelter.” (Married man, Group discussion 2)*.

*“Actually, some men want to participate just because they don’t have enough money. …They don’t have enough money to take care of the family. They are not ready to take care of many children.”(Midwife, KII 3)*.

*“…participating in FP as a man is one way of postponing your responsibilities because even if you don’t do it today tomorrow you will still have to do it. If you don’t produce children today, you will still have to produce tomorrow…. And it will be hard when you are doing what you are supposed to do 10 years back”.(Married man, Group discussion 2)*.

### Benefits of male partner involvement (n = 2)

Midwives in the key informant interviews noted that involvement of men in the use of family planning methods can reduce the incidences of gender-based violence in most families. In addition, the health workers believed that, involving men in family planning could reduce the chances of acquiring sexually transmitted infections.

*“.…. because it helps us to reduce the gender-based violence… do you know most men after these women getting involved in this family planning without their consent… it really turns out to be a bad thing in their family, you know… it really brings out violence….”(Midwife, KII 2)*.

*“Like if we encourage men also there will be a reduction in the STI because even if we have not done other methods of family planning, we tell them protection is the best way for example now the use of condoms……”(Midwife, KII 8)*.

### Perceived barriers of male involvement

The perceived barriers of male involvement in family planning included lack consent from the male partner, family planning considered a woman’s issue, work commitments, socio-cultural and religious disapproval, desire for large family size, gender roles incompatibility, misconception and fear of side effects, and health system related factors (Table 1).

### Lack of consent from the male partner (n = 8)

Male partners disapproval of family planning was perceived to discourage male partners from participating in family planning. Male partners believed that the decision to use family planning should be collectively made, but in case of disagreement, the male partner decision would stand. This was related to the male partners position as the bread winner and the head of the family who therefore was thought to reserve the right to plan for the family including determining family size. While male partners could initiate the idea to use family planning, women were accused of extramarital affairs when they expressed intentions to use family planning.

*“…every decision comes from the head of the family and that’s the man. But now if the man and the woman are not agreeing,.they go and get the services behind their man’s back…. So, such a man may not support the wife…” (Married man, Group discussion 2)*.

*“The man is the head of the family. He is the one who knows what is needed in the family. He is the one to plan for everything in the family…. But again, when the woman brings the idea of FP, then the man will be like the woman wants to cheat on me. She doesn’t want to conceive when she goes outside. That is why the man should be the first to bring the idea.” (Married man, Group discussion 1)*.

### Family planning considered a woman’s issue (n = 5)

Male partners were nonchalant about utilizing and involving them in family planning as they considered it a woman’s issue. Male partners admitted that they were less knowledgeable about family planning because of less concern for it, while others were less interested as the burden of side effects would rest on the woman.

*“They always take that FP is a woman’s thing. They always think that it is the women to do[use family planning] it.” (Midwife, KII 5)*.

*“I think the right people to be consulted are our wives because I see for us men we have less concern about that. It is the women that carry all the burden of FP” (Married man, Group discussion 2)*.

*“We normally don’t mind about this FP much or what FP is,…., just like even me, to some extent I am green about this FP ….” (Married man, Group discussion 1)*.

Some of the male partners were not willing to participate in the use of modern family planning methods as they thought they had no control over it. As such, male partners were willing to participate in family planning methods where they had control.

*“So, me I will only get involved in the things that I am able to have control over and only those that are directly to me. I will say the traditional methods, those are the ones I will participate in”(Married man, Group discussion 1)*.

### Busy work schedules (n = 4)

The nature of work particularly busy work schedules, long distance relationships and opportunity cost to spend time in the hospital were altogether perceived to hinder male participation in family planning.

*“Those ones who don’t escort maybe because….they are always engaged in their day today activities to try and provide for the family….So, what he can do is give transport money to the wife.”(Married man, Group discussion 3)*.

### Social-cultural and religious disapproval

Male involvement in family planning was greatly affected by social-cultural and religious disapproval. This occurred in form of social pressures, gender roles incompatibility, and desire for a large family size.

### Social pressure (n = 7)

Escorting the wife to the clinic and use of family planning was thought to be in complete polar opposite to societal norms as it was seen as a sign of weak manhood, submissiveness, and being remote controlled by the woman. As such, it attracted stigma to be involved in family planning. Secondly, use of family planning violated social norms that value large family size which resulted in desire to conform and bow to societal pressure. Ultimately, societal pressure discouraged male participation in family planning.

*“Now also that stigma, that the community will look at you and be like, now this guy here also who escorts the woman to the FP.”(Married man, Group discussion 2)*.

*“One, can be because of our culture and the way we were brought up… So, if I’m already tuned to that thought if you escort your woman to the clinic, you are a weak man. So, he doesn’t want to be perceived as a weak man, so definitely he cannot get involved”(Married man, Group discussion 3)*.

*“The society views those men as you know like the woman is controlling him, you know”( Married man, Group discussion 3)*.

### Religious prohibition (n = 3)

Religious beliefs which discouraged use of family planning methods in favor of many children were thought to limit male partner participation in family planning

*“Then another factor may also be the religious prohibition….a common example of the Muslim. For them, they allow you to have many wives, and that eventually means having many children and they tend to ignore those family planning methods”.(Married man, Group discussion 3, Muslim)*.

### Gender roles incompatibility (n = 7)

Male partner involvement in family planning was seen as failure of the male partner to perform the gender roles including being the head of the family. Male partners who were involved in family planning were thought to be overruled by their women and as well fear the responsibilities of having children. This compromised their position as the head of the family and made it difficult for the woman to be submissive to their authority.

*“Men should not directly participate because…you are a man; you are the one pushing your wife to go for FP…. A wise wife will look at you as a man who is fearing responsibility…. Then it will make even submission very difficult.”(Married man, Group discussion 3)*.

*“But to me, I will say, a man escorting the wife to the clinic? It means the man has totally failed to manage his own family or home…. You, have failed because how can a normal human being, a man, a real man [who] cannot think of other things other taking her to the clinic”(Married man, Group discussion 3)*.

*“Now you as a man, taking your wife to the health facility for FP, I will first ask you what has gone wrong?…… And sometimes it is true that some women over rule their men. So, they have all the authority to carry their men with them to the FP clinic and the man will just have to obey.”(Married man, Group discussion 2)*.

Although male partners were reluctant to participate in family planning, some men escorted their partners to the clinic so as to ensure that family planning has been provided especially in cases where women were reluctant to use family planning.

*“Maybe you are sure that she has not gone yet she is deceiving you. She wants maybe to produce children for you, yet for you maybe you are not ready for that child. So, you it is better you also go with her such that, that method has to go on.”(Married man, Group discussion 2)*.

### Desire for many children (n = 4)

Desire for many children was thought to hinder approval and utilization of family planning methods among male partners.

*“Some men may not want to participate, one of this is the common one, the desire to have children and you know once you have decided that you are going to have many children so automatically you may not go and use the FP” (Married man, Group discussion 2)*.

### Fear of side effects and misconceptions about male involvement

#### Fear of side effects (n = 5)

Male partners abhorred involvement in family planning because of the side effects. Infertility, vaginal bleeding, loss of libido, and congenital anomalies in children were the most feared side effects that were reported to occur from use of family planning. The male partners feared that they could be blamed for permitting their women to use family planning methods.

*“These FP methods for me I believe they can make someone not to produce, and that is why for me, personally, I don’t encourage my wife to use them…”(Married man, Group discussion 3)*.

*“……………hmmm the reason why the men don’t want to participate is that, women experience side effects, more so over bleeding…… a man finds the wife is over bleeding and asks them why and they will say they got a family planning method and that is why they are over bleeding….”(Midwife, KII 1).*

#### Misconceptions (n = 14)

Male partners had misconceptions about their involvement in the use of family planning. Some men reported that they didn’t want to participate in modern family planning methods as it encourages promiscuity and infidelity among women. Likewise, men perceived that involvement in family planning would make their partners think that they were not interested in producing children with them and could be having extramarital affairs

*“They don’t want to participate because of the most dangerous reason…. Men think that FP will open ways for this woman to start looking for other men outside. That she will start going to every man.” (Married man, Group discussion 2)*.

*“Some of the women might think that you just don’t love them and that is why you want them to go for family planning… you don’t want to produce children with them but rather go to other women……”(Married man,Group discussion* 2).

Some of the male partners did not support the use of family planning because of conspiracy theories surrounding the use of family planning methods.

*“I am negative about Uganda’s family planning to be specific because the agenda has gone wrong….it is affecting the young girls of the country….what is destroying you, you cannot be positive about it…So, me, personally, I would not encourage it. I would not encourage it.” (Married man, Group discussion 1)*.

#### Inadequate information (n = 10)

Related to misconception, inadequate information about family planning was thought to hinder male partner participation in family planning uptake.

*“I think it is because of inadequate information about FP. Some of us we really don’t know much about FP and how it is supposed to be done. That is basically some of the reasons why some men are not able to participate. Because the information is not reaching them.” (Married man, Group discussion 1)*.

### Health facility related factors (n = 5)

Health facility related factors were perceived to hinder male partner participation in family planning. Distant facilities and inaccessibility of family planning services hindered male involvement in family planning. Unconducive hospital environment including mistreatment by healthcare workers, long waiting time, and the fact that services were focused on women discouraged male participation in family planning.

*“Me, I think the first point is some of them have no access to the service. Some people are very far from the hospital and FP methods or services are not very near in these village hospitals.” (Married man, Group discussion 2)*.

*“Then the ones who don’t want to participate is one because maybe….they were talked to rudely by the nurses at the FP and they came back and said aah, I better not be there or even sometimes the men feel out of place when he is at the unit and he is the only man among so many women.” (Married man, Group discussion 2)*.

*“Aaah, actually I can say that some men just fear to go to the hospital. That is the most dangerous thing for men. Men just fear to go to the hospitals with their wives.” (Married man, Group discussion 2)*.

*“Even when you go the FP clinic ee, you as a man you feel like out of place because all this line is just full of females and you are just there, feeling out of place. The men are really uncomfortable. So, you find that as a man you are not really settled. You just keep walking up and down.” (Married man, Group discussion 1)*.

## Discussion

We explored perceptions regarding enablers and barriers to male involvement in the use of modern family planning methods in Eastern Uganda. Our study findings indicate that positive attitude and subjective norms towards family planning use, need to show support for the woman, mutual consent and limited resources encouraged male partners to be involved in the use of modern family planning methods. The barriers to male partner involvement in family planning included social, cultural and religious beliefs, misconceptions and fear of side effects, lack of male partner approval and health facility related factors.

Subjective norms and attitudes are strong predictors of behavioral intentions [17]. In our study, positive attitude regarding the benefits of family planning in general was perceived to motivate male partners to be involved in family planning. Positive attitude counteracts the prevailing hostile environment fueled by myths and misconceptions surrounding uptake and male partner involvement in family planning [18]. Our findings are consistent with studies that cite misconceptions and fear of side effects as the major deterrents for male involvement in family planning [19–21]. Subjective norms such as escorting the partner to the clinic and motivation to show support for the woman was perceived to motivate some male partners to be involved in family planning. However, negative subjective norms especially perceptions of being overruled by the woman and the associated label of a weak man deterred most men from getting involved in family planning. While escorting the woman for family planning is a good stride in uptake of modern contraception, escorting the woman could be a manifestation of gender-based violence and over controlling from male partners [22]. In our study, some of the male partners escorted their partners to the family planning clinic in order to make sure that the women received the contraception method which suggests woman’s lack of agency, autonomy, and risk for gender-based violence [23].

In study, mutual agreement as a couple was considered sine qua non for male partners to be involved in family planning [18]. Mutual consent was thought to allow partners to carefully evaluate the side effects and intentions to use family planning. Although mutual consent was valued, the male partners’ decision as the head of the family was thought to override that of the woman in cases of disagreements. The power dynamics may suggest lack of agency among women in decision making to use of family [19]. Previous studies report covert use of contraception among women to circumvent male partners disapproval of family planning use [22]. Covert contraception use against the will of the male partner is associated with increased risk of gender-based violence [22, 23].

Consistent with previous studies, male partners considered family planning a women’s issue [24]. As such, male partners were less concerned with involvement in family planning. The lack of interest for family planning manifested in form excuses of work engagements, discomfort being seen escorting the woman to the clinic and feeling out of place while in the family planning clinic. This may be related to the fact that male partners maybe ignored when in family planning clinic, while the overt mistreatment from the healthcare workers further discourages male partners from participating in family planning. Consequently, male partners were resigned to play the passive role of providing money for transport to the facility because of the unwelcoming hospital environment. Male partners play a fundamental role not just in family planning but in prevention of sexually transmitted infections [25]. Efforts to involve male partners and provide conducive welcoming hospital environment can promote participation of male partners in family planning [26].

In agrarian setting, children are highly regarded as a source of wealth, cheap labor, security and support in old age [27]. These perceptions were reinforced by underlying cultural and religious beliefs where use of family planning was prohibited in favor of many children. The desire for many children and social-cultural beliefs accounted for the underlying reservations for family planning particularly fear of infertility and promiscuity [27]. Male partners who threatened the attainment of this communal prized asset of a large family size through participating in family planning were met with social stigma, labels of weak manhood, failure to rule the family and a man who feared responsibilities [27]. These tools were powerful deterrents of male involvement as they ensured conformity to societal expectations [27]. Male partners believed that limiting the size of the family because of limited resources was untenable in the long-term. While some male partners participated in family planning because of desire to have children that they were able to provide, most of the male partners were not concerned about it [27]. Community based approaches to family planning sensitization, such as community education campaigns, may be an important step toward reducing barriers to male involvement in the use of modern family planning methods [28].

## Study strengths and limitations

The use of both group discussions and key informant interviews complemented the study findings. Particularly, the use of group discussions allowed collection of rich data as participants were able to relate thought processes of their colleagues. The group discussions were conducted by a nursing student which might have led to social desirability bias with intention of the respondents to respond in a way that they think will be acceptable and pleasing to the healthcare provider. The study findings may not be generalizable to all other settings with different cultural ethnicity. Furthermore, the group discussions only consisted of men and therefore the opinions of women on male involvement in family planning were missed out. Further studies in the same area would focus on both men and women so as to unearth multiple perspectives of the women as well as far as male involvement in the use of family planning services is concerned.

## Conclusion

Male involvement in family planning was related to the need to show support, positive attitude, subjective norms, mutual consent to participate in family planning and limited financial resources. Lack of male partner approval, social, cultural and religious beliefs, gender roles incompatibility, fear of side effects and misconceptions about male involvement in family planning, and unconducive hospital environment discouraged men from participating in family planning.

## Data Availability

The datasets used and/or analyzed during the current study are available from the corresponding author on reasonable request.

## List of abbreviations

FP: Family planning
KII: Key informant interviews
UDHS: Uganda Demographic and Health Survey
